# Case report: First report of isolated central nervous system lymphoblastic crisis in a child with chronic myeloid leukemia on dasatinib therapy

**DOI:** 10.3389/fonc.2023.1122714

**Published:** 2023-03-23

**Authors:** Suejung Jo, Jae Won Yoo, Seongkoo Kim, Jae Wook Lee, Soo-Ah Im, Bin Cho, Nack-Gyun Chung

**Affiliations:** ^1^ Department of Pediatrics, College of Medicine, The Catholic University of Korea, Seoul, Republic of Korea; ^2^ Department of Radiology, College of Medicine, The Catholic University of Korea, Seoul, Republic of Korea

**Keywords:** pediatric chronic myeloid leukemia, isolated central nervous system blastic crisis, Dasatinib, tyrosine kinase inhibitor induced optic neuropathy, cerebrospinal fluid study

## Abstract

Most children with chronic myeloid leukemia (CML) present with the chronic phase (CML-CP) at diagnosis, exhibiting an excellent treatment response to contemporary tyrosine kinase inhibitors (TKIs). However, despite TKI therapy, patients with CML-CP may progress to blastic crisis (BC). CML-BC rarely occurs in extramedullary sites, and isolated central nervous system (CNS) BC is an extremely rare condition. It may with present various neurologic symptoms that necessitates differential diagnosis from other causes such as TKI toxicity. Information on the diagnosis and treatment of this condition is lacking, as are well-established diagnostic criteria. Here, we report a case of isolated CNS lymphoblastic crisis in a child with CML-CP who was treated with dasatinib. The patient, an 8-year-old girl, was admitted owing to visual disturbance and severe headache. We highlight the importance of a CSF study for the differential diagnosis of CNS BC in patients with CML-CP who present with common neurologic symptoms during TKI therapy.

## Introduction

Chronic myeloid leukemia (CML) is rare in children and adolescents. Although pediatric patients with CML have the same cytogenetic anomaly as adults with CML, the so-called Philadelphia chromosome, which generates the *BCR::ABL1* fusion gene, pediatric CML has more aggressive clinical features and different diseases characteristics (1). Nonetheless, tyrosine kinase inhibitor (TKI) therapy yields a satisfactory treatment response in children as it does in adults. There are currently three US Food and Drug Administration-approved TKIs (imatinib, dasatinib, and nilotinib) for pediatric patients, and several clinical trials (NCT03934372, NCT04925479) for next-generation TKIs are under way.

Although survival outcomes for most patients have markedly improved with the development of TKIs, progression-free survival remains poor in patients resistant or intolerant to TKI-resistant/intolerant. In particular, once the CML-chronic phase (CML-CP) transforms into the blast phase, patient survival is poorer than *de novo* CML-blast phase, and allogeneic hematopoietic stem cell transplantation (HSCT) is the only known cure (2). The majority of cases of blastic transformation involve the bone marrow, and extramedullary blast crisis (BC) occurs as an isolated event in very few patients. The central nervous system (CNS) as a site of extramedullary BC is extremely rare, usually presenting with the clinical features of encephalitis, meningitis, or neuritis, and common neurologic symptoms such as headache, cognitive changes, increased intracranial pressure, and visual disturbance.

Here, we report a case of isolated CNS lymphoblastic crisis in a pediatric patient with CML who was previously treated with dasatinib. We highlight the importance of the differential diagnosis of CNS involvement in patients with CML presenting with common neurologic symptoms.

## Case report

An 8-year-old girl was transferred to our hospital due to hyperleukocytosis, which was incidentally discovered as she did not exhibit any symptoms. Upon physical examination, we found severe hepatosplenomegaly, with her liver and spleen palpated at about 9cm and 7cm, respectively, below the costal margin. Initial peripheral blood counts showed the following results: white blood cells (WBCs) of 198 x 10^9^/L (normal: 4-10 × 10^9^/L) with 1% blast, hemoglobin of 10.0 g/dL (normal: 11.0-14.5 g/dL), and platelets of 352 × 10^9^/L (normal: 150-450 × 10^9^/L). The bone marrow aspirate smears revealed hypercellularity with a high myeloid: erythroid ratio (higher than 20:1), and approximately 5% of all nucleated cells were lymphoblasts. Karyotyping and fluorescence *in situ* hybridization (FISH) of a bone marrow sample were positive for t(9;22)(q34;q11), leading to a diagnosis of CML-CP. The patient’s EUTOS Long-Term Survival score was 1.229, indicating that she belonged to the low-risk group (3). We started on dasatinib (60 mg/m^2^, once daily) for initial TKI therapy.

Three months after treatment, the patient exhibited both a complete hematologic response (CHR) and a complete cytogenetic response (CCyR). Quantitative polymerase chain reaction revealed a *BCR::ABL1* transcript level of 2.55% on the international scale (IS) at 3 months, indicating an optimal response. However, the therapy did not yield the milestones of an optimal response thereafter: 1.6% IS at 6 months and 0.9% IS at 12 months. Although her TKI response was at the “warning” level, we decided to maintain her on dasatinib instead of changing the medication because the *BCR::ABL1* transcript levels were constantly decreasing (**Table 1**).

**Table 1:** Cytogenetic and molecular response after dasatinib therapy according to ELN guidelines (4).

Month	Cytogenetic response	Molecular response *BCR-ABL1*% (IS)	Response assessment	Clinical events
3	Complete	2.55	Optimal	None
6	NA	1.59	Warning	None
12	NA	0.91	Warning	None
15	NA	0.32	Warning	Visual disturbance
16	Complete	0.61	Warning	Severe headache Lymphoblasts were detected in CSF

IS, international scale; CSF, cerebrospinal fluid; NA, not available.

**Figure 1:**
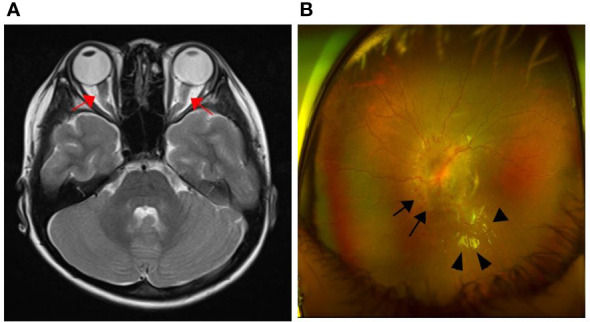
Brain MRI and fundoscopic findings at the time of visual disturbance onset. **(A)** Axial T2-weighted image showed diffuse swelling of both optic nerves (arrow); **(B)** Optic disc edema (arrow) and macular star (arrowhead).

Fourteen months after treatment, the patient was admitted due to visual disturbance. Fundoscopy revealed bilateral optic disc edema with a macular star, and brain MRI revealed diffuse swelling of both optic nerves without clear leptomeningeal and parenchymal abnormalities (**Figure 1**). Based on our initial assessment of dasatinib-induced optic neuropathy, we decided to discontinue dasatinib and start methylprednisolone pulse therapy. Within a month, her subjective symptoms and visual acuity improved. At 15 months from the start of treatment, the patient’s *BCR::ABL1* transcript level in the bone marrow remained at the warning level (0.3% IS) but had decreased from previous time points.

Two months later, the patient returned to our hospital due to severe and aggravated headaches that had persisted for two weeks. Upon examination, cerebrospinal fluid (CSF) immunophenotyping revealed a large proportion of abnormal cell populations, with approximately 89% lymphoblasts expressing CD20, TdT, and PAX-5 (negative for CD3 and MPO). The bone marrow was free of leukemic infiltration and the patient maintained a CHR and CCyR, indicating that she was experiencing an isolated CNS lymphoblastic crisis. At this time, the MRI showed an improvement in optic nerve swelling compared to the previous MRI.

**Figure 2:**
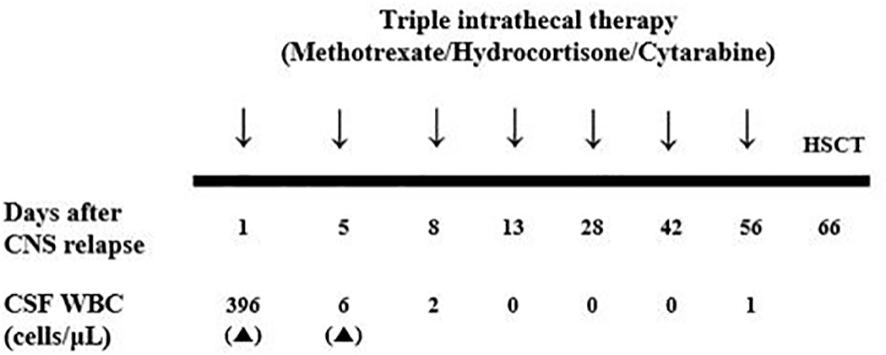
Schedule of triple intrathecal chemotherapy after CNS blast crisis and serial results of CSF WBC count. CNS, central nerve system; CSF, cerebrospinal fluid; WBC, white blood cell.

She was begun triple intrathecal (ITT) chemotherapy (methotrexate, cytarabine, and hydrocortisone) and the lymphoblast was eliminated after single dose of ITT chemotherapy. Hence, she was administered an additional five doses of ITT chemotherapy and a subsequent allogeneic HSCT was planned (**Figure 2**). Since she was still off dasatinib and had an isolated CNS relapse, we decided to resume dasatinib, which is known to penetrate the CNS, instead of switching to another TKI until allogeneic HSCT. The patient’s *BCR::ABL1* transcript level was 0.6% IS at the time of HSCT.

The patient underwent peripheral blood stem cell transplantation from a 1-allele-mismatched (HLA-A), unrelated donor. The preparative regimen consisted of myeloablative conditioning, with total body irradiation (TBI) of 1,320 cGy (from days -7 to -4), cyclophosphamide (120 mg/kg from days -3 to -2), and rabbit antithymocyte globulin (3.75 mg/kg from days -3 to -1). The infused CD34^+^-cell dose was 10.3 × 10^6^ cells/recipient kg. She developed grade 2 graft-versus-host disease in the gut, which was well tolerated with a short course of steroid treatment. Full donor chimerism was detected on day 28 post transplantation, and the *BCR::ABL1* transcript levels in the peripheral blood at 1, 3, and 6 months after transplantation were 0% IS without TKI therapy.

## Discussion

This was, to our knowledge, the first case report of an isolated CNS BC in a pediatric patient with CML on dasatinib therapy. The patient exhibited a warning response according to the European Leukemia Net 2020 criteria (4) during the TKI treatment period. As the *BCR::ABL1* transcript levels constantly decreased, we decided to continue the patient on dasatinib rather than switching to another TKI. When she experienced visual disturbance and headache, we considered two possible differential diagnoses: dasatinib-induced optic neuropathy and CNS leukemia—the most important point of our case report. Initially, we suspected that the patient had dasatinib-induced optic neuropathy rather than CNS leukemia, as dasatinib is known to be penetrate the blood-brain barrier more readily than other TKIs such as imatinib (5). Moreover, the patient’s symptoms improved after corticosteroid treatment was initiated and TKI therapy was discontinued. When she experienced another episode of severe headache, she was diagnosed with CNS leukemia after we discovered a high proportion of lymphoblasts in her CSF. Brain MRI did not reveal any specific characteristics of CNS leukemia in either episode.

As with adult patients, children with CML-CP at diagnosis typically exhibit an excellent response to contemporary TKI therapy. Dasatinib, a second-generation TKI, yielded a stronger molecular response than imatinib in a randomized clinical trial (6). However, information on progressed disease among pediatric patients on dasatinib therapy is very limited. In particular, isolated CNS relapse is an extremely uncommon condition among both adults and children. In a previous study (including both adult and pediatric patients) (7), most of the patients exhibited a CCyR at CNS relapse, and the median time from treatment initiation to CNS relapse was 29.5 (range, 3 to 112) months. All of them were treated with IT chemotherapy, CNS radiotherapy, and a switch to next-generation TKIs. In this case, the CSF lymphoblasts were eliminated after single dose of ITT therapy, and we proceeded with subsequent allogeneic HSCT after TBI-based conditioning instead of performing CNS radiotherapy and switching TKI.

Although the patient exhibited at least a sustained CCyR, she had not reached an optimal molecular response by the time of CNS relapse. Data on the correlation between *BCR::ABL1* transcript level and CNS BC are lacking. In addition, very few case reports of children and adolescents have been published, and those for whom data are available received imatinib therapy (**Table 2**). Further data is needed to determine whether the risk of CNS relapse is increased in patients with a warning response to dasatinib therapy.

**Table 2:** Clinical details of reported cases of isolated CNS relapse in pediatric chronic myeloid leukemia.

No.	Sex/age	Treatment prior to relapse	Time from diagnosis to CNS relapse	Disease status at the time of CNS relapse	Clinical presentation	Blast type	Imaging study/fundoscopy results	Treatment for CNS relapse	Reference (Year of case report)
1	M/14	Imatinib, MSD-HSCT	14 months (post-HSCT 140 days)	Complete molecular response after HSCT *(BCR::ABL1*: negative)	Headache Vomiting	Lymphoblast	NA	IT CTx. IT rituximab Cranial RT Haploidentical HSCT	(2019) Chatterjee et al. (8)
2	F/15	Imatinib	NA	CHR, CCyR *(BCR::ABL1*: NA)	Headache Vomiting Back pain	Lymphoblast	Brain MRI: thrombosis of superior sagittal sinus	IT CTx. Cranial RT Imatinib	(2011) Radhika et al. (9)
3	F/12	Imatinib	12 months	CHR, CCyR, *(BCR::ABL1*: NA)	Headache Vomiting Bilateral visual loss	Lymphoblast	Brain CT: normal Fundoscopy: optic disc edema, retinal hemorrhage	Systemic CTx. IT CTx. Switch of TKI (dasatinib) Cranial RT	(2020) Boudiaf et al. (10)
4	M/5	Untreated (parents’ preference)	8 months	CHR *(BCR::ABL1*: NA)	Headache	Lymphoblast	Brain MRI: normal	Systemic CTx. IT CTx. 2G-TKI	(2018) Jin et al. (11)
5	F/8	Dasatinib	16 months	CHR, CCyR, *(BCR::ABL1*: 0.6% [IS])	Visual disturbance Headache	Lymphoblast	Brain MRI: diffuse swelling of optic nerve Fundoscopy: optic disc edema and macular star	IT Ctx. Dasatinib HSCT	Current study

CNS, central nervous system; MSD, matched sibling donor; HSCT, hematopoietic stem cell transplantation; IT, intrathecal; CTx., chemotherapy; RT, radiotherapy; CHR, complete hematologic response; CCyR, complete cytogenetic response; IS, international scale; MRI, magnetic resonance imaging; CT, computed tomography; TKI, tyrosine kinase inhibitor; 2G, second-generation; NA, not available.

Although ocular toxicity secondary to TKI therapy, which is characterized by mild periorbital and macular edema, has been described, it is a relatively rare and self-limited adverse effect. Fewer cases of dasatinib- and nilotinib-induced ocular events have been reported (we are aware of only one case report of optic neuropathy secondary to dasatinib) (12). With the lack of well-established diagnostic criteria for ocular toxicity after TKI, diagnoses must be made according to the patient’s clinical symptoms. In this case, the patient’s first episode of visual disturbance was reduced after initiation of corticosteroid therapy and discontinuation of dasatinib therapy. A limitation of our case report is that we do not know whether the first episode (which we assessed as dasatinib-induced optic neuropathy at that time) was related to CNS leukemia or dasatinib toxicity. Both the first and second MRIs in this report did not reveal any clear leptomeningeal or parenchymal abnormalities, which are the most specific findings for CNS involvement in leukemia. We observed only a thickening of the optic nerve, which may result from either leukemic infiltration or optic neuropathy. In particular, there have been a few previous case reports regarding dasatinib, which have shown atypical neurologic symptoms without other specific MRI findings (12, 13). Additionally, previous methylprednisolone pulse therapy for treating initial visual symptoms may have masked key findings in the second MRI. Although we were unable to clearly distinguish between the two causes based on the MRI findings, this suggests that MRI alone may be insufficient for differential diagnosis. A CSF study conducted during the first episode would have been more helpful in distinguishing between dasatinib-induced optic neuropathy and CNS BC-related symptoms.

Genetic studies using next-generation sequencing have revealed additional genetic abnormalities in CML beyond *BCR::ABL1* mutation, which can drive disease progression and drug resistance (14). Commonly mutated genes include *ASXL1, RUNX1, IZKF1, BCORL1, KMT2D, TP53*, and *DNMT3A.* Mutations in epigenetic modifier, such as *ASXL1*, can predict drug resistance in CML, demonstrating the potential of genetic mutation-based risk stratification (15). Although genetic screening is not currently included in the guidelines, growing data on larger cohorts will provide evidence to future clinical practice for managing CML.

## Conclusion

This case highlights that, although rare, an isolated CNS relapse should be considered in patients with CML on dasatinib therapy who experience common neurologic symptoms such as visual disturbance and severe headache. A comprehensive approach, including both a CSF study and brain MRI, is needed for prompt diagnosis.

## Data availability statement

The original contributions presented in the study are included in the article/supplementary material. Further inquiries can be directed to the corresponding author.

## Ethics statement

Written informed consent was obtained from the minor(s)’ legal guardian/next of kin for the publication of any potentially identifiable images or data included in this article.

## Author contributions

Conceptualization, SJ and JY. Methodology, SJ, JY, and N-GC. Validation, SJ, JY, and N-GC. Formal analysis, SJ and JY. Investigation, SJ and JY. Data curation, SJ, JY, S-AI, N-GC. Writing—original draft preparation, SJ and JY. Writing— review and editing, SJ, JY, SK, and JL. Supervision, N-GC and BC. All authors have read and agreed to the published version of the manuscript.
